# Iridescence in Meat Caused by Surface Gratings

**DOI:** 10.3390/foods2040499

**Published:** 2013-11-11

**Authors:** Juan Leonardo Martinez-Hurtado, Muhammad Safwan Akram, Ali Kemal Yetisen

**Affiliations:** Department of Chemical Engineering and Biotechnology, University of Cambridge, Tennis Court Road, Cambridge CB2 1QT, UK; E-Mail: msa40@cam.ac.uk

**Keywords:** meat, muscle tissue, iridescence, drying, diffraction grating, quality

## Abstract

The photonic structure of cut muscle tissues reveals that the well-ordered gratings diffract light, producing iridescent colours. Cut fibrils protruding from the muscle surface create a two-dimensional periodic array, which diffract light at specific wavelengths upon illumination. However, this photonic effect misleads consumers in a negative way to relate the optical phenomenon with the quality of the product. Here we discuss the fundamentals of this optical phenomenon and demonstrate a methodology for quantitatively measuring iridescence caused by diffraction gratings of muscle tissue surface of pork (*Sus scrofa domesticus*) using reflection spectrophotometry. Iridescence was discussed theoretically as a light phenomenon and spectral measurements were taken from the gratings and monitored in real time during controlled drying. The findings show that the intensity of diffraction diminishes as the surface grating was dried with an air flow at 50 °C for two minutes while the diffracted light wavelength was at 585 ± 9 nm. Our findings indicate that the diffraction may be caused by a blazed surface grating. The implications of the study include providing guidelines to minimise the iridescence by altering the surface microstructure, and in consequence, removing the optical effect.

## 1. Introduction

Iridescence is an optical phenomenon in which the light is diffracted from a photonic structure at a specific wavelength or a rainbow-like colour [1]. The iridescent colours are not caused by pigments, but by the interference of light with the morphology of the structure [2,3]. These photonic structures comprise of periodically ordered materials (or air gaps) with varying refractive index. The interference of light with the structure and its scatter causes a photonic effect in which only a dominant wavelength or multiple wavelengths are scattered back. Photonic structures including surface gratings, multilayers and thin films have been observed in nature [4] on the surface tissues or cuticle of a variety of species including birds [5,6], insects [7], marine molluscs [8] and plants [9]. The diversity in photonic structures might provide camouflage, warning colouration, superiority in reproductive behaviour, signal communication, thermoregulation and conspecific recognition. An analogous, but non-evolution-driven, photonic effect is also noticed in meat cuts, which display a rainbow-like multicoloured iridescence. Because it is difficult to distinguish the photonic effect from the original absorption colour of the meat, consumers may associate this phenomenon with spoilage, chemical or bacterial contamination, which may lead to refusal of the meat products. Considering the importance of this optical phenomenon in meat industry and costumer misconception, there is a need to investigate the factors causing meat iridescence to take preventive measures to minimise consumer concerns [10].

Muscular tissues are formed by elongated cells in a regular arrangement showing a well-ordered structure [11]. When viewed at specific angles, such photonic structures may produce iridescence. The orientation and spacing of the ordered structures are different from muscle to muscle and type of species; the iridescence, if present, is also likely to be different. For instance, different types of muscle tissues could produce different colours and intensities. It has been found that fish tissue slices produce colours that vary depending on the species and environmental conditions [12]. Muscle fibres in mammals are supported by structures formed by myofilament proteins grouped in fibrils that form cell fibres; when cross-sectioned at a certain angle, the diffracted light may produce iridescence depending on the structure. The arrangement of the cells in the whole muscle may have a different order and/or orientation depending on the muscle tissue type, however, it is usually homogenous. In fact, certain cuts of pork and beef exhibit iridescence [13,14], while others do not. In the case of beef, iridescence is most frequently found in semitendinosus muscle; it has a homogenous size distribution of cells and cylindrical fibrils that form a regular grid in transverse sections [15]. Several studies have investigated the association of iridescence with physiological factors such as pH, phosphate concentration, maturity, cooking temperature, fat thickness, surface roughness, shear force, animal type and sex type [16,17]. Along with these factors, iridescence in muscle tissue can be directly associated with water content. Since water is the main composition of the cell, changes in water content can cause evident changes in structure or optical properties [18]. Thus, the water content can be related to other factors like pH or ionic strength [19]. Therefore, characterisation of the structure and optical properties of the materials is crucial for understanding iridescence in natural structures. Previous studies have shown that iridescence in muscle tissue appears at a wavelength range seen as green, orange or red colours [20]. In recent investigations of cooked beef *iliocostalis* [21] and yellow fin tuna (*Thunnus albacares*) [22], multilayer interference has been suggested as an explanation for the diffracted colours with strong angular dependence. In this work, we investigate the iridescence of cooked pork muscle tissue and report the intensity of the diffracted light in real time during controlled drying. We show that the intensity of iridescence is dependent on the water content of the tissue and the diffraction angle.

## 2. Experimental Section

**Figure 1:**
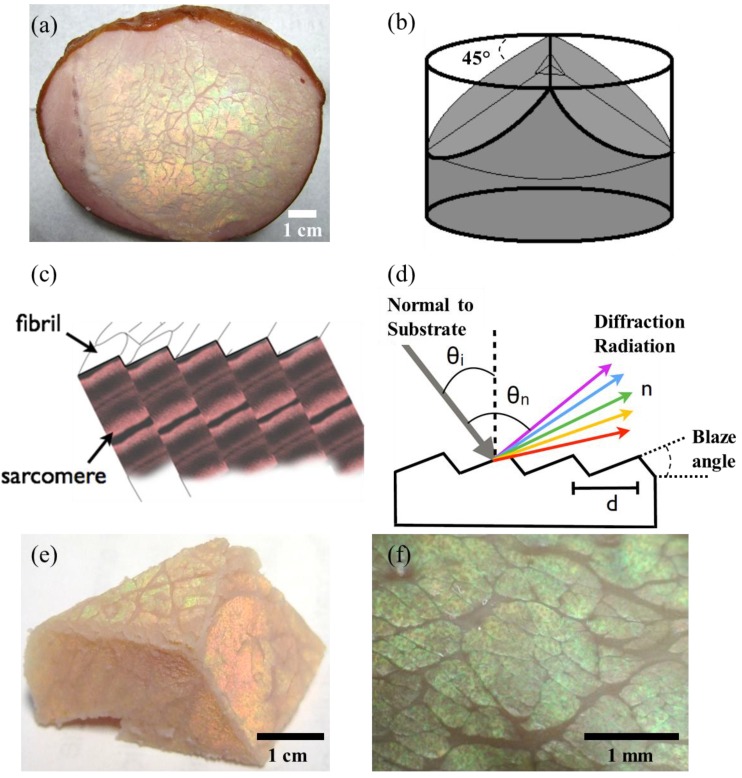
Muscle tissue of a pork loin sample displays iridescence when viewed at a specific angle. (**a**) The loin muscles sliced transversely to the long axes of muscle fibres. (**b**) Section planes of muscle tissue prepared for observing fibres from different angles. (**c**) A schematic of the periodicity of muscle fibres and fibrils. (**d**) The light interference with a diffraction grating comprising of a constant refractive index and periodicity. (**e**) A pyramidal cut of the muscle tissue showing the angular dependence of iridescence colours, and the prevalence of the iridescence along the sample. (**f**) Microscopic images of the surface of the muscle tissue illuminated at the angle *θ_i_* to produce the maximum diffraction.

A commercial pork (*Sus scrofa domesticus*) loin sample was supplied by the commercial processing facility (Morliny Sopocka, Animex, Warsaw, Poland). The same loin muscle tissue was used throughout the measurements along with the control sample from the same cut. The loin muscles were sliced transversely to the long axes of muscle fibres using a sharp blade (Figure 1a), and then it was carved from the centre at 45° to show the prevalence of the iridescence along the muscle tissue (Figure 1b). The resulting cut, exposed the muscle fibrils at the surface cross section (Figure 1c), exposing regular striations (sarcomere, Z-line), which resemble blazed gratings (see scanning electron micrographs of reference [11]). The micrographs were acquired with a 400× USB microscope back-illuminated with LED light (2.0 MP camera). The full scale photographs were taken with a digital SLR camera (Canon EOS 400D). Light diffracted from a blazed grating can be expressed theoretically, considering the surface of the muscle tissue is illuminated with a light source at an angle *θ_i_* (Figure 1d). The intensity of the diffracted light will have a maximum for a particular wavelength obeying the grating equation [23]:

*n* × *λ* = *d* × (sin*θ_n_* + sin*θ_i_*) (1)

where *n* is the order of diffraction (*n* = 1), *λ* is the diffracted wavelength, *d* is the grating period, *θ_n_* is the angle of observation, and *θ_i_* is the angle of incidence.

Diffraction measurements were recorded with a spectrophotometer (AveSpec 2028, Avantes, Apeldoorn, The Netherlands) running on AvaSoft^©^ (v7.5.3) with 100 ms integration time. The detector was placed in a fixed position facing the muscle tissue, and the muscle tissue was illuminated with a continuous focused (*d* = 3 mm) white light source at the angle of maximum diffraction intensity. *θ_n_* and *θ_i_* were kept constant throughout the readings. *λ_max_* values were recorded at time intervals of 3 s (450–850 nm). A hot air flow at 50 °C (default setting of the heat gun) was used to dry the surface of the sample while measuring the diffracted wavelength as described elsewhere [24].

The gratings present on the surface of the muscle tissue display colour diffraction at different angles of view (or illumination), suggesting that the structure is a well-ordered surface grating rather than a multilayer structure. Tilting the viewing angle caused a change in colour and intensity observed due to both the diffraction grating and the background colour, which also causes a back scattering. Figure 1e illustrates the muscle tissue when viewed at different angles; the upper side of the pyramidal cut diffracts yellow/green whereas the front side diffracts orange-red due to the angular dependence. Figure 1f is the microscopic images of the surface of the muscle tissue illuminated at the angle *θ_i_* to produce the maximum diffraction. The colour displayed was yellow-green-, which corresponds to the wavelength of ~585 nm.

## 3. Results and Discussion

**Figure 2:**
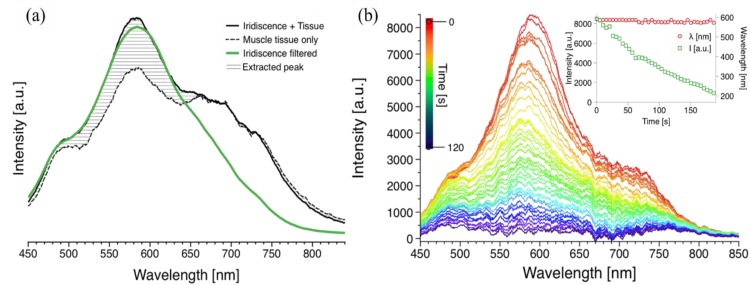
Diffraction spectra of the muscle tissues for static and real-time measurements (**a**) continuous line shows the raw spectral measurement at the angle of incidence with maximum diffraction intensity; dashed line corresponds to the colour of the muscle tissue (absorption); and green line represents the filtered diffraction spectrum. (**b**) Real-time measurement of diffraction of the diffraction grating during drying. Each plot was recorded every 3 s; the top spectrum (red) shows the diffraction intensity at *t* = 0 s, and the bottom spectrum (violet) represents the diffraction intensity at *t* = ~2 min. The inset represents the steady diffraction wavelength and the decreasing intensity of the diffraction peak.

The background colour of the muscle tissue interferes strongly with the measurements. Figure 2a shows the measured spectra of the sample: the dashed plot corresponds to the muscle tissue colour only, the continuous line shows the diffraction at the angle of maximum incidence (*λ_max_*), and the green line represents the same peak using a near-infrared filter for the diffraction from the muscle tissue. The spectral noise due to the hue of the muscle tissue was subtracted, resulting in the spectra in Figure 2b at *t* = 0. The hatched area is the intensity gain due to the diffraction.

During drying the muscle tissue was fixed at the angle of incidence (*θ_i_*) with maximum diffraction and the sample was exposed to an air flow at 50 °C. The mass of a dried material is *w* = *W* × [*X*/(1 + *X*)], where *W* is the mass of the material being dried and *X* is the moisture content freely available on the surface [24]. For a constant rate of drying as in our case, *w* × (d*X*/d*t*) = constant, we observed a linear decay in the intensity of the diffraction peak, which is directly affected by structural changes caused by the variation in the water content [19]. There was no discoloration of the tissue sample, hence only evident moisture removal. The initial wavelength was stable with a peak value of 585 ± 9 nm and it did not deviate from this value. The spectral response was recorded every 3 s; the intensity of the original diffraction peak in red (top of graph) diminished over time (~2 min) as represented by the different colours (Figure 2b). The intensity decreased from ~8500 light counts (arbitrary units) to less than ~500 light counts as shown by the time dependent plots in Figure 2b inset, corresponding to the maximum diffraction (peak) values. The removal of water through drying the surface of the muscle tissue, may result in compact fibres, and hence a surface with no iridescence. Additionally, the diffracted peak wavelength during the drying was also measured in real time; no substantial change was recorded in the diffraction peak value (Figure 2b inset). This suggests that the exposed meat structure due to its angular dependency has the characteristics of a surface grating rather than a multilayer structure [25]. A control experiment was also performed to corroborate the stability of the iridescence, the values of wavelength and intensity. The intensity values for this initial control measurement were also stable for a constant value of ~8000 light counts, comparable to the initial brightness of the sample subjected to drying.

## 4. Conclusions

Iridescence is a structure dependent light phenomenon that is influenced by the optical properties of the muscle tissues, such as refractive index, that are directly related to the physiological factors such as water content. The surface of the muscle tissue consists of quasi regularly oriented and spaced bundles of fibres, which comprise of well-ordered myofibrils. When the fibres are sliced, fibrils protrude from the surface of the muscle tissue, which creates a periodic array which acts like a diffraction grating [11]. Therefore, upon illumination, light waves scattered by the well-ordered protrusions and microfibrils constructively interfere and diffract light at specific wavelengths. We quantified the diffraction from pork loin muscle tissues with real-time spectrophotometry during drying. Drying the surface of an iridescent muscle tissue sample, thus, removing water from the constituent fibres caused the intensity of the diffraction to vanish while the diffracted wavelength remained stable. This phenomenon is a result of a change in refractive index and/or surface structure due to the decrease in water content. In contrary to the analogous evidence provided for the muscle tissues of beef *iliocostalis* [21] and yellow fin tuna (*Thunnus albacares*) [22], our data indicates the presence of a blazed surface grating in pork (*Sus scrofa domesticus*). Our evidence is based on the diffraction wavelength variability upon viewed from different angles, the intensity decrease of the diffraction intensity and the stability of the diffraction wavelength during the drying. The data showed that the intensity of the diffracted light at the peak decreased to 1% of the total initial diffraction, which corresponded to the iridescence only, and not the tissue color background. However, future studies should establish detailed angular dependence and analysis of the predicted photonic structure through spectrophotometry and electron microscopy, respectively. A practical conclusion of this study is that short periods (~2 min) of surface drying can be used to minimise diffraction, and thus iridescence. The findings of this study will contribute to the fundamental understanding of spectrophotometric analysis of meat for quality assessment of water (moisture) content and toughness.
